# Supplementary material for: Effect of PM2.5 Levels on ED Visits for Respiratory Causes in a Greek Semi-Urban Area
Source: J Pers Med. 2022 Nov 5;12(11):1849. doi: 10.3390/jpm12111849 (PMC9696598; doi:10.3390/jpm12111849)
Supplement: Supplementary file 1 [file jpm-12-01849-s001.zip › jpm-1867968-supplementary.pdf]

Supplementary: Table S1

URI	$PM_{2.5}$ Level	Total Days	Number of ED Visits	Mean ED Visits/Day ±SD	% Increase in Mean ED Visits	P value
Winter	<25 µg/m3	18	15	0.83±0.92	51.23%	0.22
	≥25 µg/m3	73	92	1.26±1.05		
Spring	<25 µg/m3	42	36	0.86±1.12	-16.00%	0.54
	≥25 µg/m3	50	36	0.72±1.03		
Summer	<25 µg/m3	74	38	0.51±1.01	19.01%	0.70
	≥25 µg/m3	18	11	0.61±0.70		
Autumn	<25 µg/m3	54	45	0.83±1.01	20.00%	0.44
	≥25 µg/m3	37	37	1.00±1.00		

Table S2

Pneumonia	$PM_{2.5}$ Level	Total Days	Number of ED Visits	Mean ED Visits/Day±SD	% Increase in Mean ED Visits	P value
Winter	<25 μg/m3	18	7	0.39±0.50	76.13%	0.85
	≥25 μg/m3	73	50	0.68±0.98		
Spring	<25 μg/m3	42	33	0.79±1.28	-5.82%	0.85
	≥25 μg/m3	50	37	0.74±1.03		
Summer	<25 μg/m3	74	28	0.38±0.74	-26.59%	0.61
	≥25 μg/m3	18	5	0.28±0.75		
Autumn	<25 μg/m3	54	25	0.46±0.86	-0.76%	0.98
	≥25 μg/m3	37	17	0.46±0.77		

Table S3

Asthma exacerbation	$PM_{2.5}$ Level	Total Days	Number of ED Visits	Mean ED Visits/Day±SD	% Increase in Mean ED Visits	P value
Winter	<25 µg/m3	18	1	0.06±0.24	23.29%	0.50
	≥25 µg/m3	73	5	0.07±1.25		
Spring	<25 µg/m3	42	15	0.36±0.62	-38.40%	0.23
	≥25 µg/m3	50	11	0.22±0.47		
Summer	<25 µg/m3	74	8	0.11±0.39	-48.61%	0.59
	≥25 µg/m3	18	1	0.06±0.24		
Autumn	<25 µg/m3	54	6	0.11±0.32	45.95%	0.49
	≥25 µg/m3	37	6	0.16±0.37		

Table S4

COPD exacerbation	$PM_{2.5}$ Level	Total Days	Number of ED Visits	Mean ED Visits/Day±SD	% Increase in Mean ED Visits	P value
Winter	<25 µg/m3	18	9	0.50±0.86	-26.03%	0.18
	≥25 µg/m3	73	27	0.37±0.70		
Spring	<25 µg/m3	42	18	0.43±0.77	30.67%	0.41
	≥25 µg/m3	50	28	0.56±0.73		
Summer	<25 µg/m3	74	28	0.38±0.66	-11.90%	0.79
	≥25 µg/m3	18	6	0.33±0.49		
Autumn	<25 µg/m3	54	21	0.39±0.60	80.69%	0.15
	≥25 µg/m3	37	26	0.70±0.94		
